# The systemic exercise-released chemokine lymphotactin/XCL1 modulates *in vitro* adult hippocampal precursor cell proliferation and neuronal differentiation

**DOI:** 10.1038/s41598-019-48360-5

**Published:** 2019-08-14

**Authors:** Odette Leiter, Stefanie N. Bernas, Suse Seidemann, Rupert W. Overall, Cindy Horenburg, Susann Kowal, Gerd Kempermann, Tara L. Walker

**Affiliations:** 1 Center for Regenerative Therapies Dresden (CRTD), Technische Universität Dresden, 01307 Dresden, Germany; 2 German Center for Neurodegenerative Diseases (DZNE) Dresden, 01307 Dresden, Germany; 3 Present Address: Queensland Brain Institute, The University of Queensland, Brisbane, 4072 Australia

**Keywords:** Adult neurogenesis, Neural stem cells, Neuroimmunology

## Abstract

Physical exercise has well-established anti-inflammatory effects, with neuro-immunological crosstalk being proposed as a mechanism underlying the beneficial effects of exercise on brain health. Here, we used physical exercise, a strong positive modulator of adult hippocampal neurogenesis, as a model to identify immune molecules that are secreted into the blood stream, which could potentially mediate this process. Proteomic profiling of mouse plasma showed that levels of the chemokine lymphotactin (XCL1) were elevated after four days of running. We found that XCL1 treatment of primary cells isolated from both the dentate gyrus and the subventricular zone of the adult mice led to an increase in the number of neurospheres and neuronal differentiation in neurospheres derived from the dentate gyrus. In contrast, primary dentate gyrus cells isolated from XCL1 knockout mice formed fewer neurospheres and exhibited a reduced neuronal differentiation potential. XCL1 supplementation in a dentate gyrus-derived neural precursor cell line promoted neuronal differentiation and resulted in lower cell motility and a reduced number of cells in the S phase of the cell cycle. This work suggests an additional function of the chemokine XCL1 in the brain and underpins the complexity of neuro-immune interactions that contribute to the regulation of adult hippocampal neurogenesis.

## Introduction

Adult hippocampal neurogenesis refers to the formation of functional new neurons in a brain region which is essential for learning and memory. The regulation of this process, however, is complex and much still remains to be understood. Neural precursor cells (NPCs) in the subgranular zone (SGZ) of the hippocampal dentate gyrus (DG) are responsive to environmental cues, with physical activity being one of the strongest positive physiological modulators. The increase in exercise-induced NPC proliferation^1^ is associated with changes in the systemic environment. We have recently shown that treatment with serum samples from mice that ran for only four nights increased the number of neurospheres in primary DG cultures, suggesting that neurogenesis-promoting factors were present in the blood of the running mice^2^. Peripheral growth factor changes have been studied following exercise, revealing increased levels of circulating vascular endothelial growth factor^3^, insulin-like growth factor-1^4^, β-endorphin^5^ and serotonin^6^, all of which have been shown to be required for the increased NPC proliferation following exercise in rodents^3,4,6,7^. Furthermore, cathepsin b, a myokine secreted from skeletal muscle into the blood stream in response to exercise, has been associated with the running-induced formation of new neurons^8^. However, it is likely that circulating immune factors also contribute to the modulation of hippocampal neurogenesis following physical exercise. Despite physical separation by the blood brain barrier, considerable neuro-immune crosstalk occurs between the circulatory system and cells of the hippocampal stem cell niche under physiological conditions^9^. Moreover, transcriptomic analysis of isolated populations of NPCs from the DG has revealed that neural stem cells have immune cell-like characteristics^10^, indicating the potential regulation of these cells by immune factors. Although the exact communication pathways between NPCs and immune cells are unknown, the major regulatory molecules of intercellular communication within the immune system, cytokines and chemokines, can also be recognized by neural cells^11^. Recent work in the ageing field shows that chemokines can influence adult hippocampal neurogenesis^12^, and it is known that NPCs express several chemokine receptors^13^. One example that underpins the importance of chemokine signalling in the neurogenic niche is stromal cell-derived factor 1-mediated signalling, which promotes the proliferation^14^ and maturation^15^ of NPCs, as well as the maintenance of the neural stem cell pool in the DG^16^. Moreover, the platelet-derived chemokine platelet factor 4, which is released into the blood following exercise, was recently shown to stimulate neurogenesis, particularly neuronal differentiation^2^. In the present study, we used proteomic profiling to identify additional immune factors capable of mediating exercise-induced adult neurogenesis. We found that the chemokine lymphotactin (XCL1) was increased in the plasma of running versus sedentary mice and investigated whether XCL1 contributes to the regulation of adult hippocampal NPCs.

## Results

### XCL1 plasma levels increase in mice following 4 days of exercise

**Figure 1:**
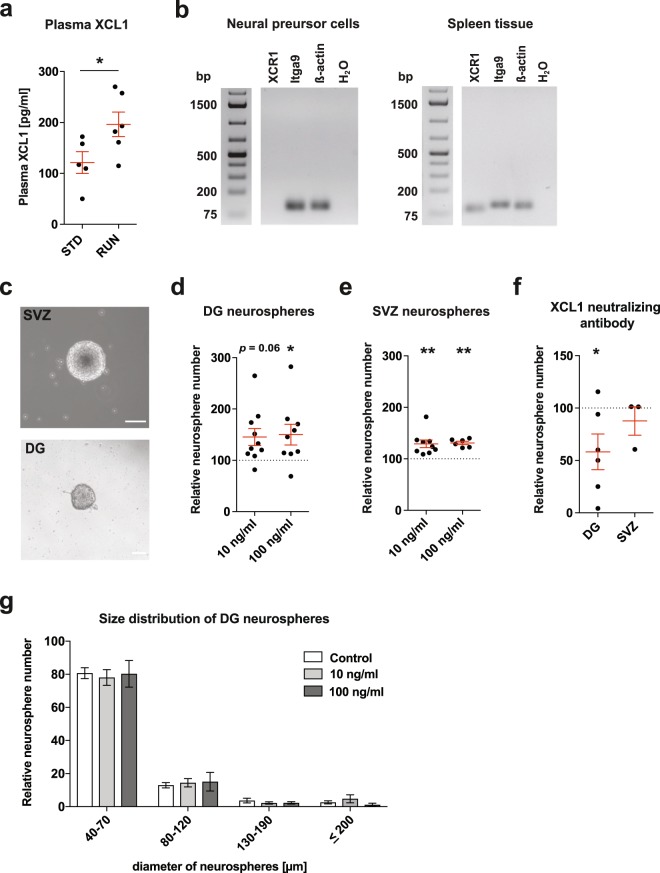
XCL1 plasma levels rise after running and XCL1 treatment increases the number of neurospheres. (**a**) XCL1 plasma levels measured by an ELISA in standard-housed mice (STD, n = 5 mice) and mice housed for 4 days with a running wheel (RUN, n = 6 mice). *p < 0.05, Student’s *t*-test. (**b**) qPCR gene expression analysis of lymphotactin receptors reveals that neural precursor cells express *Itga9* but not *Xcr1* (left), although both *Itga9* and *Xcr1* are detected in splenic control tissue (right). Uncropped gels are presented in Supplementary Fig. S1. (**c**) Representative images of a SVZ neurosphere (top) and a DG neurosphere (bottom). Scale bars: 100 μm. (**d**) Neurosphere assays with DG-derived primary cells cultured with XCL1. n = 9 to 10 independent experiments, *p < 0.05, one-way ANOVA with Dunnett test. (**e**) Neurosphere assays with SVZ-derived primary cells cultured with XCL1. n = 6 to 9 independent experiments, **p < 0.01, one-way ANOVA with Dunnett test. (**f**) Neurosphere assays with XCL1-neutralizing antibodies. n = 3 to 6 independent experiments, *p < 0.05, one-way ANOVA with Dunnett test. (**g**) Size distribution of DG-derived neurospheres cultured with XCL1. n = 7 to 8 independent experiments. Dashed lines represent control cultures normalized to 100%. All data represent the mean ± SEM.

Physical exercise is a strong positive mediator of adult hippocampal neurogenesis. To identify systemic immune-related proteins that could potentially affect adult neurogenesis via neuro-immune crosstalk, we performed custom mouse multiplex immunoassays on plasma isolated from sedentary mice (STD) and mice housed in cages with access to a running wheel for 4 days (RUN). We determined the plasma levels of 66 intercellular signalling molecules, including cytokines, chemokines, complement factors and growth factors that are involved in intercellular communication (see Supplementary Table S1). These data indicated a higher plasma concentration of the chemokine XCL1 in RUN versus STD control animals (see Supplementary Table S1). Using an enzyme-linked immunosorbent assay (ELISA) to quantify protein concentrations in plasma, we confirmed the RUN-induced increase in XCL1 following 4 days of exercise in a second cohort of mice (RUN 196.4 ± 24.0 pg/ml vs STD 121.3 ± 21.4 pg/ml, *t*_9_ = 2.79, *p* = 0.048; Fig. 1a).

### XCL1 increases the number of neurospheres

To investigate whether NPCs have the potential to respond to XCL1, we first examined whether NPCs express a receptor for this chemokine. In peripheral tissue, XCR1 has long been the only known receptor for XCL1^17^. However, a recent publication identified integrin alpha subunit 9 (Itga9) as an alternative receptor^18^. To determine which of these receptors is expressed by NPCs, a quantitative real-time polymerase chain reaction (qPCR) was performed on DG-derived NPCs. Both *Itga9* and *Xcr1* were expressed in spleen tissue, which was used as a positive control (Fig. 1b). However, only *Itga9* was expressed in the NPCs, suggesting that XCL1 could act on these cells through the Itga9 receptor (Fig. 1b).

Given that XCL1 plasma levels are significantly elevated following exercise, we next aimed to determine whether NPCs from the two major neurogenic niches in the brain are responsive to increases in XCL1. For this, primary cells were isolated from both the DG and the subventricular zone (SVZ) and cultured as neurospheres (Fig. 1c) in the presence of different concentrations of XCL1. In this assay, the number of neurospheres serves as a proxy readout for the effect of the XCL1 treatment on the number of actively proliferating NPCs in each culture. In a pilot experiment we tested five different XCL1 concentrations ranging from 100 pg/ml to 250 ng/ml (see Supplementary Fig. S2). We found that NPCs responded to the XCL1 treatment and determined 10 ng/ml and 100 ng/ml to be the most effective doses in the neurosphere cultures. We therefore chose to perform all subsequent experiments at these concentrations and found approximately 50% more neurospheres in XCL1-treated DG cultures (10 ng/ml: 145.4 ± 16.5% of control, *p* = 0.06; 100 ng/ml: 150.1 ± 20.2% of control, *p* = 0.04; Fig. 1d) and approximately 30% more neurospheres in SVZ cultures supplemented with XCL1 (10 ng/ml: 129.2 ± 7.4% of control, *p* = 0.004; 100 ng/ml: 130.9 ± 3.2% of control, *p* = 0.005; Fig. 1e and Supplementary Tables S2 and S3). To determine whether the effect of XCL1 could be blocked, we then performed neurosphere assays with XCL1-neutralizing antibodies and observed a significant reduction in the number of neurospheres in DG-derived cultures (58.1 ± 17.0% of control, *p* = 0.047; Fig. 1f), further confirming the ability of XCL1 to regulate NPC activity.

As physical exercise is known to primarily stimulate NPC proliferation in the DG^1,19^, we also determined the size of the DG neurospheres, which provides information about the proliferative capacity of each neurosphere-forming cell. For this, the diameter of the neurospheres was measured and the neurospheres were then classified into different size categories. We found that the size distribution of the DG neurospheres cultured with XCL1 was not significantly different from that in control cultures, suggesting that XCL1 has no direct effect on the proliferative capacity of NPCs (Fig. 1g).

### XCL1 promotes neuronal differentiation *in vitro*

**Figure 2:**
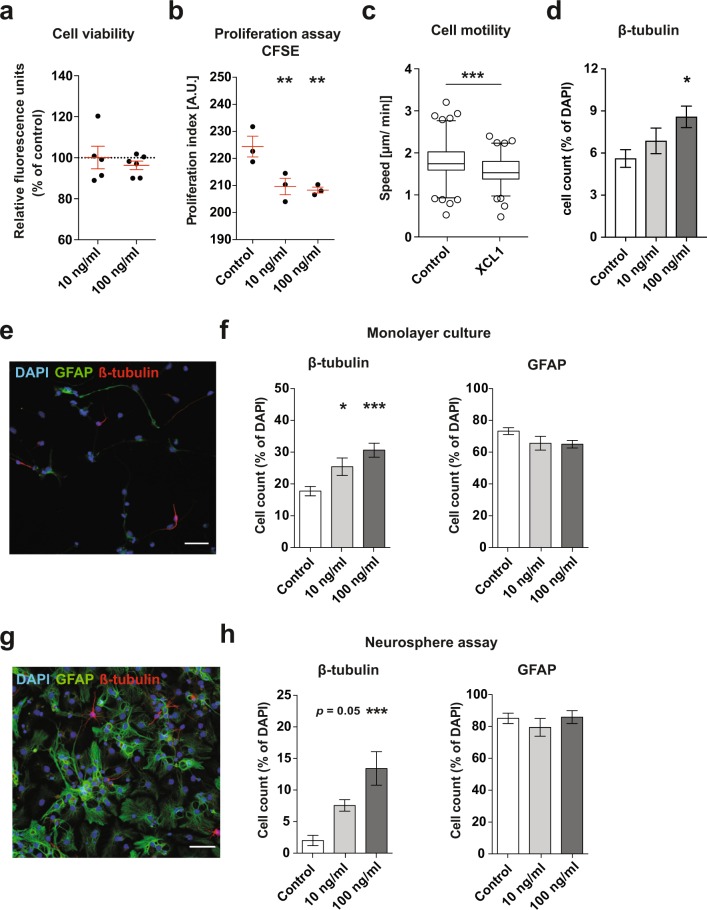
XCL1 promotes neuronal differentiation in adherent monolayer and neurosphere cultures. (**a**) Viability assay in adherent NPC cultures with XCL1. n = 5 to 6 independent experiments. (**b**) CFSE proliferation assay in adherent NPC cultures with XCL1. n = 3 independent experiments, **p < 0.01, one-way ANOVA with Dunnett test. (**c**) Motility of adherent monolayer-cultured NPCs determined by semi-automated tracking. Data are plotted as the 5^th^/95^th^ percentile with outliers represented as circles. XCL1: n = 92 cells, Control: n = 104 cells, ***p < 0.001, Student’s *t*-test. (**d**) Quantifica*t*ion of β-tubulin^+^ cells in proliferating NPC cultures two days after the addition of XCL1. n = 4 independent experiments, *p < 0.05, one-way ANOVA with Dunnett test. (**e**) Representative image of differentiated NPCs in adherent monolayer cultures showing GFAP^+^ astrocytes in green and β-tubulin^+^ neurons in red. Scale bar: 50 μm. (**f**) Quantification of GFAP^+^ and β-tubulin^+^ cells in differentiated adherent monolayer cultures treated with XCL1. n = 4 to 5 independent experiments, *p < 0.05, ***p < 0.001, one-way ANOVA with Dunnett test. (**g**) Representative image of differentiated neurospheres showing GFAP^+^ astrocytes in green and β-tubulin^+^ neurons in red. Scale bar: 50 μm. (**h**) Quantification of GFAP^+^ and β-tubulin^+^ cells in differentiated neurosphere cultures treated with XCL1. n = 5 independent experiments, ***p < 0.001, one-way ANOVA with Dunnett test. Dashed lines represent control cultures normalized to 100%. All data represent the mean ± SEM.

To investigate the effects of XCL1 treatment on DG-derived NPCs in more detail, we next performed experiments in more homogeneous NPC adherent monolayer cultures. Using a resazurin reduction cell viability assay, we first excluded the possibility of any potential toxic effects of XCL1 on NPCs in culture, observing no difference in the number of viable cells following XCL1 treatment (Fig. 2a). In order to address NPC proliferation, the cells were then cultured under proliferation conditions with growth factors for two days in the presence of XCL1, after which a CFSE proliferation assay was performed. This revealed a significant reduction in the number of proliferating cells in the cultures treated with XCL1 (10 ng/ml: *p* = 0.02; 100 ng/ml: *p* = 0.01; Fig. 2b), suggesting that the cells might be starting to differentiate in the presence of XCL1. Previous findings suggest that the migration speed of NPCs *in vitro* indicates the prospective differentiation of the cells, with neuronal differentiation being associated with a lower migration efficiency^20^ and the differentiation into astrocytes correlating with higher motility (our unpublished data). To study cell motility of proliferating NPCs cultured as monolayers with and without 100 ng/ml XCL1, we used semi-automated tracking and followed the cells for 3.5 days using automated time-lapse microscopy. The tracking data revealed that the cells indeed moved at a significantly slower rate in XCL1-treated cultures (XCL1: 1.5 ± 0.04 μm/min vs control: 1.8 ± 0.05 μm/min, *t*_194_ = 3.78, *p* = 0.0002; Fig. 2c), indicating that the NPCs could already be biased towards neuronal differentiation. To further investigate this possibility, we examined neuronal differentiation in proliferating NPC cultures two days after the addition of XCL1, the same time point at which the CFSE analysis was performed and the cells were tracked. Consistent with the above assumption, we found that more cells expressed β-tubulin, indicating that XCL1 promoted the differentiation towards neurons, even when the cells were cultured under proliferation conditions (% of total cells, control: 5.6 ± 0.6%; 10 ng/ml: 6.8 ± 0.9%; 100 ng/ml: 8.6 ± 0.7%, *p* = 0.04; Fig. 2d).

We next performed a differentiation assay in which adherent NPCs were cultured for four days without growth factors, allowing their differentiation into glia and neurons. We again found a significant increase in the number of β-tubulin^+^ neurons in cultures supplemented with XCL1 (% of total cells, control: 17.7 ± 1.5%; 10 ng/ml: 25.4 ± 2.8%, *p* = 0.04; 100 ng/ml: 30.6 ± 2.2%, *p* = 0.0006; Fig. 2e,f). In addition, we assessed the number of GFAP^+^ astrocytes in the cultures and found that the increase in β-tubulin^+^ cells did not occur at the expense of cells converting to the astrocyte lineage (Fig. 2f). Similarly, when we differentiated neurospheres cultured in the presence of XCL1 for seven days, we found significantly more β-tubulin^+^ neurons (% of total cells: control: 2.0 ± 0.8%; 10 ng/ml: 7.6 ± 1.0%, *p* = 0.05; 100 ng/ml: 13.4 ± 2.7%, *p* = 0.0001), whereas the number of GFAP^+^ cells remained unaffected (Fig. 2g,h). Together, these data indicate a robust effect of XCL1 on neuronal differentiation.

### XCL1 influences the cell cycle progression of neural precursor cells *in vitro*

**Figure 3:**
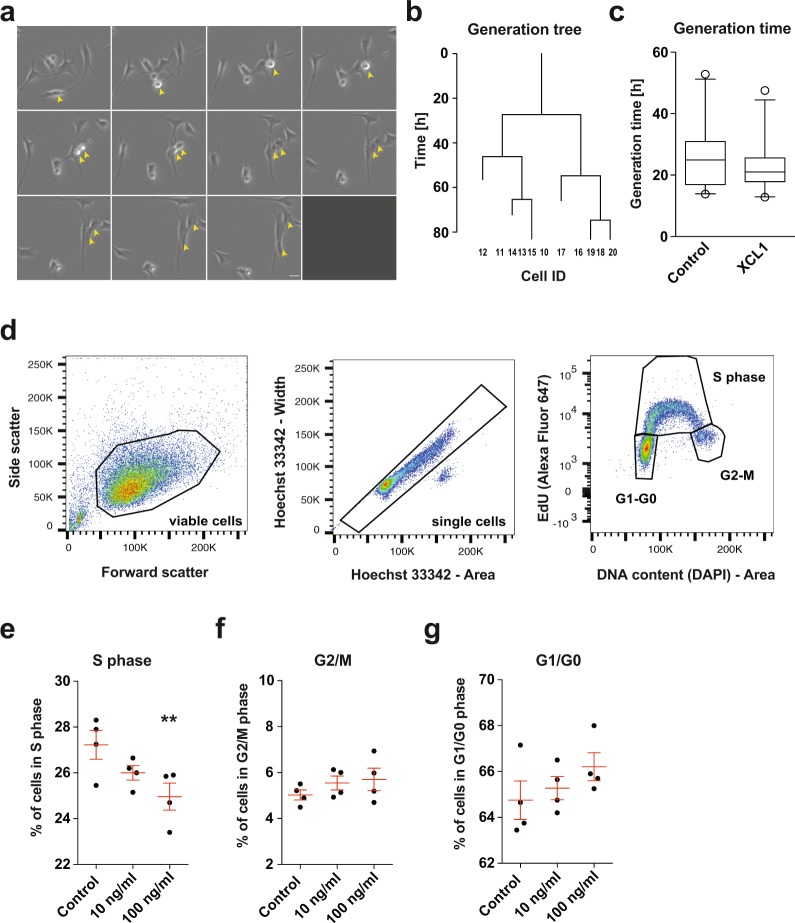
XCL1 influences the cell cycle progression of NPCs *in vitro*. (**a**) Representative images of a dividing NPC followed by time-lapse microscopy. Images are 5 min apart. Yellow arrows mark the process of cell division. Scale bar: 10 μm. (**b**) Example of a generation tree of a re-dividing cell obtained from semi-automated cell tracking of NPCs to calculate the mean generation time. (**c**) Generation time of NPCs cultured with and without XCL1 determined by semi-automated cell tracking. Data are plotted as the 5^th^/95^th^ percentile with outliers represented as circles. Control: n = 23 cells, XCL1: n = 26 cells. (**d**) Representative flow cytometry plots of the click-iT EdU proliferation assay. Viable cells were first defined using forward scatter and side scatter (left). Doublets were then excluded from single cell signals by plotting Hoechst-width against Hoechst-area (middle). Finally, to determine the cell cycle phase, the DNA content (Hoechst label) was plotted against the EdU signal (right). (**e**) Percentage of NPCs in S phase. n = 4 independent experiments, **p < 0.01, one-way ANOVA with Dunnett test. (**f**) Percentage of NPCs in G2/M phases. n = 4 independent experiments. (**g**) Percentage of NPCs in G1/G0 phases. n = 4 independent experiments. All data represent the mean ± SEM.

It is known that neuronal differentiation in the adult hippocampus is associated with cell cycle changes, with more committed NPCs having a shorter S phase and a shorter total cell cycle length than proliferating progenitor cells with higher proliferative potential^21–23^. Given that more cells differentiate into neurons in XCL1-treated cultures, we next investigated the cell cycle dynamics of proliferating NPCs in adherent monolayer cultures supplemented with XCL1. We first used semi-automated tracking to determine the generation time of NPCs cultured with and without 100 ng/ml XCL1 (Fig. 3a,b). This analysis revealed that the average length of the cell cycle was about 3 h shorter in cultures supplemented with XCL1 (22.6 ± 1.5 h) compared with that of cells in vehicle-treated control cultures (25.8 ± 2.1 h), indicating that XCL1-treated cells cycle faster (Fig. 3c).

We then performed a click-iT EdU assay to investigate the distribution of cells in the S phase, G2/M phases or G1/G0 phases of the cell cycle (Fig. 3d). We found that the number of cells in S phase in cultures treated with XCL1 decreased in a dose-dependent manner, being statistically significant at 100 ng/ml (control: 27.2 ± 0.6%; 10 ng/ml: 26.0 ± 0.3%; 100 ng/ml: 24.9 ± 0.6%, *p* = 0.03; Fig. 3e), whereas the distribution of cells in the G2/M and G1/G0 phases was not affected (Fig. 3f,g). This is in line with our differentiation data and indicates that the cell cycle shortens when XCL1 is present.

### XCL1 knockout mice have lower *ex vivo* neurogenesis

**Figure 4:**
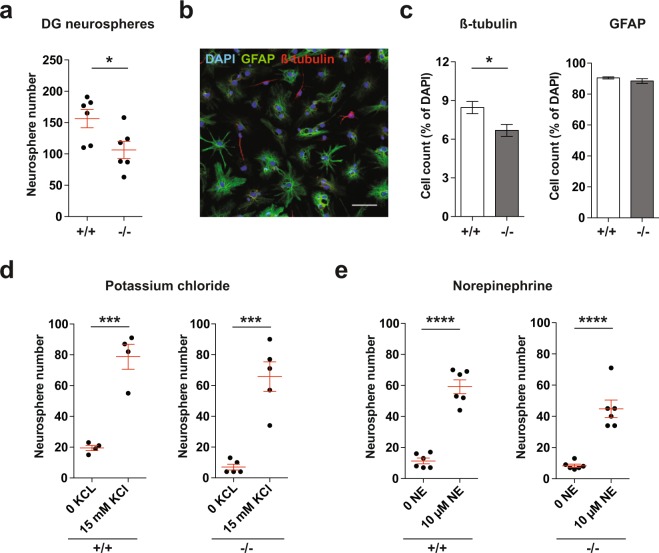
Neurogenesis in XCL1 KO mice is reduced *ex vivo*. (**a**) Neurosphere assays with primary DG cells from XCL1 KO mice (−/−) and WT littermates (+/+). n = 6 mice per group, *p < 0.05, paired Student’s *t*-test. (**b**) Representative image of differentiated neurospheres showing GFAP^+^ astrocytes in green and β-tubulin^+^ neurons in red. Scale bar: 50 μm. (**c**) Quantification of GFAP^+^ and β-tubulin^+^ cells in differentiated neurosphere cultures from XCL1 −/− and +/+ mice. n = 4 mice per group, *p < 0.05, Student’s *t*-test. (**d**) and (**e**) Neurosphere assays with primary DG cells from XCL1 −/− and +/+ mice in the presence of (**d**) potassium chloride (n = 4 to 5 independent experiments) and (**e**) norepinephrine (n = 6 independent experiments). ***p < 0.001, ****p < 0.0001, Student’s *t*-test.

Having shown that XCL1 treatment influences NPC activity, we next determined whether the lack of XCL1 has an effect on NPCs. For this, we used XCL1 knockout (KO; −/−) mice, which lack functional *Xcl1* mRNA and protein through a homozygous KO in the *Xcl1* gene^24^. To address NPC proliferation and potential *ex vivo*, we isolated primary cells from the DG of age- and sex-matched XCL1 −/− mice and their wildtype littermates (WT; +/+) and performed neurosphere assays. Two WT-KO pairs were females and four were males. We found significantly fewer neurospheres in all cultures from XCL1 −/− mice compared with their +/+ littermate controls (106 ± 14 neurospheres vs 156 ± 15 neurospheres, *t*_5_ = 2.9, *p* = 0.03; Fig. 4a). Following quantification, the neurospheres were differentiated (Fig. 4b). Although the number of GFAP^+^ cells was similar between groups, XCL1 −/− mice had significantly fewer β-tubulin^+^ cells than their +/+ littermates (% of total cells: 6.6 ± 0.5% vs 8.5 ± 0.5%, *t*_6_ = 2.7, *p* = 0.04; Fig. 4c), indicating that the lack of XCL1 could also cause a decline in neurogenesis *ex vivo*.

To investigate whether the reduction in neurosphere number in XCL1 −/− cultures could be rescued by additional stimulation, we supplemented neurosphere cultures from XCL1 −/− and +/+ mice with either potassium chloride (KCl) or norepinephrine. Both treatments have been shown to activate latent NPCs, leading to an increased number of neurospheres^25,26^. In KCl-supplemented cultures, we found a significant increase in the number of neurospheres in cultures from both XCL1 +/+ (79 ± 8 vs 20 ± 2 neurospheres, *t*_6_ = 7.1, *p* = 0.0004) and XCL1 −/− mice (66 ± 10 vs 7 ± 2 neurospheres, *t*_8_ = 6.1, *p* = 0.0003; Fig. 4d). Similarly, norepinephrine treatment significantly increased the neurosphere number in cultures from both genotypes (XCL1 +/+: 59 ± 5 vs 11 ± 2 neurospheres, *t*_10_ = 9.9, *p* < 0.0001; XCL1 −/−: 45 ± 6 vs 8 ± 1, *t*_10_ = 6.4, *p* < 0.0001; Fig. 4e). These results indicate that even though fewer neurospheres formed *ex vivo* from XCL1 −/− mice (Fig. 4a), the number of resident latent NPCs that could be activated was similar in both +/+ mice and those lacking the chemokine.

Together these data suggest a role of XCL1 in the regulation of adult hippocampal neurogenesis, with XCL1 exerting pro-neurogenic effects on NPCs, in particular promoting their differentiation into neurons.

## Discussion

In this study we investigated whether adult hippocampal neurogenesis can be mediated by crosstalk between immune molecules and NPCs. Using physical exercise as a model of increased neurogenesis, we identified the chemokine XCL1 as a candidate factor that may contribute to this effect.

Although XCL1 has not been previously linked to adult neurogenesis, other work has emphasized the importance of chemokine signalling in the regulation of NPCs in the hippocampal stem cell niche^12,15,16^. XCL1 represents an unusual type of chemokine, in that it lacks two of the four cysteine residues that are typical of all other chemokine families^27^, making it an interesting candidate to exert functions other than chemoattraction through the XCR1 receptor^24^. A recent study which reported anti-apoptotic effects of XCL1 on skeletal muscle cells^28^ underpins the alternative actions of this chemokine. Although expression of the classical XCL1 receptor *Xcr1* could not be detected in our NPC cultures, the recently identified XCL1 receptor Itga9^18^ was present, providing a potential alternative for XCL1-mediated signalling in these cells. Integrins are known to be expressed in brain tissue and on neural stem cells^29–33^; however, Itga9 expression on NPCs has not been previously reported. The study of Matsumoto *et al*.^18^ was the first to demonstrate a direct interaction between a chemokine and an integrin, which could be a potential route of interaction between chemokines and cells, including NPCs.

We found that the NPCs only responded to supraphysiologic doses of XCL1 *in vitro*, suggesting that the chemokine is unlikely to contribute to *in vivo* neurogenesis under baseline conditions. Although the concentrations of XCL1 used in the present study are higher than physiological plasma levels, the fact that NPCs are responsive to the chemokine indicates that they are equipped with the necessary machinery to enable this interaction. Therefore, XCL1 could act on NPCs in conditions where XCL1 levels substantially rise, such as after immune-related responses. Upon microglial activation, these cells upregulate the XCL1 receptor and also secrete XCL1^34^, which could result in local XCL1 increases within the neurogenic niche. Moreover, although the *in vitro* model does not resemble the *in vivo* neurogenic niche, where other cells or co-factors are present, the NPCs could respond to different concentrations *in vivo*. Using XCL1 neutralizing antibodies, we found a significant reduction in the number of neurospheres formed from primary DG tissue, suggesting that XCL1 was present in the culture medium. Although we have not investigated whether NPCs themselves secrete XCL1, microglia, which are known to secrete XCL1^34^ and are also present in the initial preparation of the primary tissue, are a likely source of the XCL1 antagonized by the neutralizing antibody in the neurosphere cultures.

In the periphery, XCL1 is primarily secreted into the blood stream by activated CD8^+^ T cells^35–37^, and the number of CD8^+^ T cells increases after physical exercise^38,39^, indicating a link to the increased levels of plasma XCL1 observed following exercise. It has also been reported that XCL1 promotes the T helper cell type (Th) 1 response^40^, and that peripheral Th1 responses are enhanced by physical exercise^41,42^. Such immune responses can be sensed by the hippocampal stem cell niche and have been shown to promote neurogenesis^43–45^, indicating a potential link between physical activity, raised XCL1 plasma levels and an increase in hippocampal neurogenesis.

Although physical activity is thought to primarily stimulate the proliferation of NPCs^1,19^, a plethora of exercise-induced changes regulates the process of neurogenesis along the trajectory from a stem cell to a fully functional mature neuron, with the systemic chemokine XCL1 appearing to contribute to this complex regulation. Our data demonstrate that XCL1 alone has pro-neurogenic effects on NPCs *in vitro*, particularly promoting their differentiation into neurons. This was supported by our finding that cells supplemented with XCL1 had shorter cell cycle lengths, in particular a shorter S phase, which is known to be a characteristic of NPCs during neuronal differentiation^23^. These data suggest that XCL1 may stimulate neurogenic processes at later stages of running-induced neurogenesis, such as the differentiation, maturation or survival of NPCs, rather than the initial proliferative response. This is in accordance with our finding that XCL1 KO mice generated fewer DG-derived neurospheres and displayed reduced neuronal differentiation, although the number of resident latent NPCs was not different from that of WT mice, as measured after stimulation with KCl and norepinephrine. In line with the assumption that the effects of XCL1 are not primarily pro-proliferative, we found no elevated XCL1 plasma levels after one night of running (data not shown). In contrast, a previous study reported increased NPC proliferation in the DG following only a single night of exercise^46^, indicating that additional factors likely stimulate such a response. This accords with the results of the proteomic profiling of mouse plasma from running and non-running mice that we performed in another study, which identified a number of additional systemic changes that occur following acute periods of exercise^2^.

XCL1 has been shown to act together with other cytokines as a functional unit in the Th1 immune reaction^40^, including interferon (IFN)-γ, which has been shown to promote neurogenesis^47,48^. Although IFN-γ was not included in our proteomic analysis, it has previously been shown that IFN-γ levels are elevated after acute moderate exercise in peripheral blood mononuclear cells^42^, indicating a response to exercise. This suggests that XCL1 could play different functional roles in combination with additional co-factors that were not investigated here. The effects of IFN-γ on NPCs were mediated by microglia^47^, which survey their surroundings in order to detect and respond to environmental changes^49^. Bidirectional crosstalk between other peripheral immune factors and microglia has been previously suggested^50^. A recent study reported that the cells in primary microglial cultures respond to XCL1 treatment and express the XCL1 receptor XCR1, with its expression being up-regulated after microglial activation^34^. Microglia, the immune cells within the brain, are also responsive to exercise^51–53^ and the density of microglia in the DG inversely correlates with adult hippocampal NPC proliferation^53^. Moreover, microglia have been shown to mediate exercise-induced increases in NPC proliferation via signalling through the microglial receptor C-X3-C motif chemokine receptor 1 (CX3CR1) and the neural cell-released chemokine CX3CL1^54^, further suggesting that chemokine signalling is an important mediator within the neurogenic niche. Recent work from Kettenmann and colleagues showed that the XCR1 receptor is expressed in adult hippocampal microglia, but not in adult microglia isolated from the cortex^55^, suggesting a possible XCL1-dependent role of microglia in adult neurogenesis. Whether the exercise-increased levels of XCL1 we observed alter microglia number and function, and whether this could be a possible route for the regulation of NPCs following exercise, remain to be determined.

Although XCL1 had a pro-neurogenic effect on NPCs *in vitro*, we propose that extrinsic cues, such as acute exercise, result in the release of a cocktail of systemic factors that work in concert, rather than in isolation, to promote NPC proliferation and neurogenesis *in vivo*. By promoting cell cycle exit and neuronal-lineage differentiation, we suggest that pro-neurogenic factors, such as XCL1, support the effect of pro-proliferative factors to achieve the desired functional outcome — increased adult neurogenesis.

## Materials and Methods

### Mice

Mice were housed on a 12 h light/dark cycle with water and food provided *ad libitum*. Female C57BL/6JRj mice were purchased from Janvier Labs, France. Homozygous XCL1 knockout mice^24^ were kindly provided by Richard Kroczek (Robert Koch Institut, Berlin) and maintained as a heterozygous breeding colony at the animal facility of the Center for Regenerative Therapies. All mice were 8 weeks old at the beginning of the experiment. All animal experiments were conducted with approval from the local ethics committee (Landesdirektion Sachsen) and in accordance with the European and national regulations (Tierschutzgesetz).

### Blood collection and plasma preparation

Female C57BL/6JRj mice were housed individually for 4 nights with or without a running wheel (TSE Systems). In the morning of day 5, the mice were anaesthetized with ketamine (100 mg/kg)/xylazine (20 mg/kg) in 0.9% sodium chloride, and whole blood was collected transcardially into EDTA-coated tubes. The blood was centrifuged twice at 2000 × g for 15 min and the plasma stored at −80 °C for no longer than 2 weeks.

### Multi-analyte profiling and enzyme-linked immunosorbent assay

Samples from five mice per group (standard housing or running) were analysed by Myriad RBM™ using the 66-biomarker multi-analyte profiling RodentMAP^®^ v3.0. Protein levels were also measured using the mouse XCL1 PicoKine™ ELISA Kit (Boster Biological Technology), according to the manufacturer’s instructions. The plate was read at 450 nm using a microplate reader within 30 min of addition of the stop solution.

### Quantitative real-time PCR

Total RNA was isolated from cells of a DG-derived NPC line and from the spleen of 8-week-old female C57BL/6JRj mice using the RNeasy mini kit (Qiagen). 1 µg of total RNA was reverse transcribed with random hexamer primers using the Superscript II Reverse Transcriptase kit (Invitrogen). Quantitative real-time PCR was performed using the QuantiFast SYBR Green PCR kit (Qiagen). Each cDNA sample (100 ng/μl) was amplified using a Bio-Rad CFX Connect™ Real-Time System cycler, with primers specific for *Xcr1* (Xcr1_fw 5′-GCACTGGAGGAGATCAAAGG-3′ and Xcr1_rev 5′-CGGGATGCAGGGATACTGAG-3′) or *Itga9* (Itga9_fw 5′-ATGACGGGTTCCCAGATG-3′ and Itga9_rev 5′-TGTAGACTGCGCCAGCAA-3′). β-actin was used as an endogenous control (β-actin_fw 5′-TGACCCAGATCATGTTTGAGA-3′ and β-actin_rev 5′-GGAGAGCATAGCCCTCGTAG-3). Reactions with dH_2_O in the absence of cDNA were included as a negative control for PCR amplification.

### Neurosphere assay

DG and SVZ primary cells were isolated from the brains of C57BL/6JRj and XCL1 KO mice as described previously^56^. The SVZ or DG from both hemispheres of “n” mice (where n is the number of conditions to be tested) were diluted in n × 20 ml of neurosphere growth medium (neural basal medium containing 0.5% B-27^®^ supplement (50X), 0.25% penicillin/streptomycin (10,000 U/ml) and 0.25% GlutaMAX™ (100X)), supplemented with epidermal growth factor (EGF; 20 ng/ml), fibroblast growth factor-2 (FGF-2; 20 ng/ml) and heparin (2 μg/ml). Cells were then seeded at 200 μl per well across 96-well plates so that the final plating density corresponded to the cells of the DG or SVZ of one mouse per plate. Compounds, including recombinant mouse XCL1 protein (R & D Systems) and mouse XCL1 antibodies (5 μg/ml; R & D Systems) diluted in 0.1% bovine serum albumin (BSA) in phosphate-buffered saline (PBS), were added immediately before seeding. Control cultures were treated with the respective volume of 0.1% BSA in PBS. For neurosphere assays with potassium chloride (15 mM) and norepinephrine (10 μM), the cells were cultured as previously described^25,26^ in DMEM/F-12 basal medium containing mouse NeuroCult NSC Proliferation Supplements, 0.2% BSA, 0.25% penicillin/streptomycin (10,000 U/ml) and 0.25% GlutaMAX™ (100X) supplemented with EGF (20 ng/ml) and FGF-2 (20 ng/ml). After 7 days (SVZ) or 12 days (DG), the neurospheres were counted and their diameter measured. Quantification was performed blinded to the experimental groups. Results are expressed as relative neurosphere number, which equates to counts per condition normalized to the value for a vehicle-treated control of the same experiment. The raw counts of each neurosphere experiment are presented in Tables S2 and S3.

For differentiation, approximately 15 neurospheres were randomly selected and plated onto poly-D-lysine (PDL; 5 μg/ml) and laminin (5 μg/ml)-coated coverslips in a 24-well plate containing neurosphere growth medium without growth factors. After 7 days, the adherent cells were washed with PBS and fixed with 4% paraformaldehyde (PFA) in PBS.

#### Immunostaining of differentiated neurospheres

Coverslips with fixed cells were washed with PBS before blocking was performed for 1 h in blocking solution (PBS containing 10% normal donkey serum (NDS) and 0.2% Triton X-100). Incubation with primary antibodies against glial fibrillary acidic protein (GFAP; 1:500; Agilent) and β-III-tubulin (1:2000; Promega) diluted in antibody solution (PBS containing 3% NDS and 0.2% Triton X-100) was performed for 1 h. After washing with PBS, the cells were incubated with appropriate fluorochrome-conjugated secondary antibodies for 30 min. Within the final four washing steps, the cells were incubated with DAPI (1:2000) for 10 min. The coverslips were dip-washed in dH_2_O and mounted onto glass slides. Quantification of differentiated cells was performed on a total of 20 random fields of view (FOV), from two coverslips per experiment. The FOV were selected unbiased in the DAPI channel. Cells were imaged using a Zeiss AxioImager Z1 microscope with ApoTome attachment and AxioVision Rel. 4.8 software. Images were acquired at 200 x magnification. β-III-tubulin^+^ cells, GFAP^+^ cells and DAPI^+^ cells were counted using Photoshop^®^ CS6 Version 13.0 × 6. The percentages of differentiated cells per experiment were determined by quantifying the total number of β-III-tubulin^+^ cells and GFAP^+^ cells relative to the total number of DAPI-labelled cells. An average of approximately 1200 cells were analysed per coverslip.

### Adherent monolayer culture

A NPC line was generated from the DGs of adult C57BL/6JRj mice as previously described^56^. Unless otherwise indicated, NPCs from an 80% confluent culture were seeded at a density of 2 × 10^4^ cells/cm^2^ into PDL/laminin-coated wells with or without coverslips and cultured in proliferation medium (neural basal medium containing 0.5% B-27^®^ supplement (50×), 0.25% penicillin/streptomycin (10,000 U/ml) and 0.25% GlutaMAX™ (100X), 20 ng/ml EGF and 20 ng/ml FGF-2) for 48 h. Recombinant mouse XCL1 protein (R & D Systems) was reconstituted at 100 μg/ml stock solution in 0.1% BSA in PBS. Control cultures were supplemented with 0.1% BSA in PBS.

#### Differentiation assay of neural precursor cells

Differentiation of adherent monolayer cells was performed as described earlier^56^. After 48 h, the proliferation medium was removed and replaced by growth medium containing 5 ng/ml FGF-2 but no EGF, as well as recombinant mouse XCL1 protein or 0.1% BSA. After 48 h the medium was replaced by growth medium without growth factors and supplements, and the cells were cultured for four days. On day nine, the differentiated cells were fixed with 4% PFA in PBS. Immunostaining and quantification of differentiated monolayer cells were performed on two coverslips per condition as described for differentiated neurospheres. An average of approximately 300 cells were analysed per coverslip.

#### Resazurin viability assay of neural precursor cells

NPCs were cultured under proliferation conditions with recombinant mouse XCL1 or 0.1% BSA. After 48 h, the medium was replaced by fresh proliferation medium containing resazurin in dH_2_O at a final concentration of 15 μg/ml and the cells were incubated for 2 h at 37 °C. Fluorescence values were measured using a microplate reader with an excitation wavelength of 535 nm and an emission wavelength of 590 nm. The mean fluorescence was calculated for each culture condition from three technical replicates. The mean measured fluorescence of three cell-free reference wells was used to determine the background.

#### CFSE proliferation assay of neural precursor cells

Proliferation of NPCs from an 80% confluent culture was measured using the CellTrace™ CFSE Cell Proliferation Kit (ThermoFisher Scientific) according to the manufacturer’s instructions. In brief, 2.5 × 10^6^ cells were resuspended in PBS containing 1% BSA and incubated with CFSE (7.5 μM in DMSO) for 10 min at 37 °C. The cells were then quenched with ice-cold PBS containing 1% BSA and incubated for 5 min on ice. Following centrifugation at 300 × g for 5 min, one third of the resuspended cells was fixed as a positive control. The remaining cells were seeded into 6-well plates at a density of 2 × 10^4^ cells/cm^2^, and the wells were supplemented with recombinant mouse XCL1 protein or 0.1% BSA. Two technical replicates were performed per experiment. After 48 h, a medium change was performed without further addition of XCL1. After two more days, the cells were fixed with 4% PFA in PBS. Per condition, a total of 20,000 CFSE-labelled cells were analysed using an LSRII flow cytometer. The median fluorescence intensity (MFI) of the CFSE dye was measured using a 488 nm laser and a 530/30 bandpass filter. The raw data were analysed using Flow Jo. MFI values were normalized to the positive control. The proliferation index was calculated as 1 divided by the normalized MFI value.

#### Click-iT^®^ EdU Flow Cytometry Cell Proliferation Assay of neural precursor cells

NPCs were cultured in proliferation medium with recombinant mouse XCL1 protein or 0.1% BSA. After 48 h, the Click-iT^®^ EdU Flow Cytometry Cell Proliferation Assay (Thermo Fisher Scientific) was performed according to the manufacturer’s instructions. Subsequently, analysis was performed using an Aria III flow cytometer (BD Biosciences) and the FlowJo software. In total, the experiment was performed four times with two technical replicates each.

#### Semi-automated tracking of neural precursor cells

NPCs from an 80% confluent culture were seeded at a density of 0.5 × 10^4^ cells/cm^2^ into PDL/laminin-coated wells of a 24-well glass bottom plate (Greiner Bio-One) and cultured under proliferation conditions with recombinant mouse XCL1 protein or 0.1% BSA. Cell tracking was performed by time-lapse microscopy using an inverted, motorized spinning disk microscope (Zeiss Axio Observer.Z1) with a fully integrated incubation chamber to control temperature (37 °C) and CO_2_ (5%). Images were acquired every 5 min with 20 ms exposure time, using a Zeiss EC Plan-Neofluar 10 × 0.3 Ph1 objective. Semi-automated (user-supervised) analysis was performed using CellTracker^57^, CellProfiler^58^ and R (version 3.1.2; CRAN; R Core Team, 2014). The images are first segmented and cells automatically tracked with CellProfiler^58^. A self-written R script then transcribed the data for a user-based correction step, which was performed using CellTracker^57^.

### Statistical analysis

All analyses were performed using Graph Pad Prism 6 or R/Bioconductor. All data are represented as mean ± SEM. Unless otherwise indicated, comparison of two groups was performed using unpaired, two-tailed student’s *t*-tests with assumed equal variances. When comparing more than two groups, a one-way ANOVA with *post hoc* Dunnett comparison was performed. Conventional statistical significance was set at *p* < 0.05.

## Supplementary information

Supplementary information

## Data Availability

The datasets generated during and/or analysed during the current study are available from the corresponding author on reasonable request.
