# Supplementary material for: The systemic exercise-released chemokine lymphotactin/XCL1 modulates in vitro adult hippocampal precursor cell proliferation and neuronal differentiation
Source: Sci Rep. 2019 Aug 14;9:11831. doi: 10.1038/s41598-019-48360-5 (PMC6694144; doi:10.1038/s41598-019-48360-5)
Supplement: Supplementary file 1 — Supplementary information [file 41598_2019_48360_MOESM1_ESM.pdf]

Supplementary information

The systemic exercise-released chemokine lymphotactin/XCL1 modulates *in vitro* adult hippocampal precursor cell proliferation and neuronal differentiation

Odette Leiter[1,2,3], Stefanie N. Bernas[1,2], Suse Seidemann[1], Rupert W. Overall[1,2], Cindy Horenburg[1], Susann Kowal[1], Gerd Kempermann[1,2] and Tara L. Walker*[1,2,3]

[1] Center for Regenerative Therapies Dresden (CRTD), Technische Universität Dresden, 01307 Dresden, Germany

[2] German Center for Neurodegenerative Diseases (DZNE) Dresden, 01307 Dresden, Germany

[3] current address: Queensland Brain Institute, The University of Queensland, Brisbane 4072, Australia

Supplementary Table S1: Plasma proteomic profiling revealed several changes in protein levels in response to exercise. Relative plasma concentrations (mean ± SEM) in standard-housed mice and mice after 4 days of running (n = 5 individual 8-week old female C57BL/6JRj mice per group) measured by custom mouse multiplex immunoassays (data of all 66 measured molecules; Rules based medicine, USA). The *p*-values represent the results of *t*-tests with Benjamini and Hochberg correction.

**Analyte**	**Units**	**Standard housing**	**Running wheel**	***p*-value**
Apolipoprotein A-I (Apo A-I)	[µg/ml]	214.6 ± 15.1	218.4 ± 13.3	0.86
C-Reactive Protein Mouse (CRP)	[µg/ml]	5.56 ± 0.5	7.46 ± 1.1	0.14
CD40 (CD40)	[pg/ml]	33.2 ± 1.4	32 ± 1.5	0.58
CD40 Ligand (CD40-L)	[pg/ml]	1,350 ± 87.8	1,139 ± 83.2	0.12
Eotaxin	[pg/ml]	1,081 ± 59.8	1,014 ± 82.2	0.53
Epidermal Growth Factor Mouse (EGF)	[pg/ml]	51 ± 2.1	44.6 ± 1.4	* 0.04
Factor VII	[ng/ml]	63.6 ± 2.7	61.4 ± 3.3	0.62
Fibrinogen	[µg/ml]	7,675 ± 755.5	3,969.6 ± 1,047	* 0.03
Fibroblast Growth Factor 9 (FGF-9)	[ng/ml]	<2.9	<2.9	NA
Fibroblast Growth Factor basic (FGF-basic)	[ng/ml]	<15	<15	NA
Granulocyte Chemotactic Protein-2 Mouse (GCP-2)	[ng/ml]	18 ± 0.6	18.6 ± 2.7	0.83
Granulocyte-Macrophage Colony-Stimulating Factor (GM-CSF)	[pg/ml]	<6.8	<6.8	NA
Growth Hormone (GH)	[ng/ml]	25.8 ± 13.9	7.8 ± 2.4	0.30
Growth-Regulated Alpha Protein (KC/GRO)	[ng/ml]	0.07 ± 0.01	0.05 ± 0.008	0.21
Haptoglobin	[µg/ml]	126.2 ± 0.8	125.8 ± 0.8	0.74
Immunoglobulin A (IgA)	[µg/ml]	87.4 ± 16.5	93.2 ± 19.8	0.83
Insulin	[uIU/ml]	<7.5	<7.5	NA
Interferon gamma (IFN-γ)	[pg/ml]	<38	<38	NA
Interferon gamma Induced Protein 10 (IP-10)	[pg/ml]	97.6 ± 10.4	87 ± 10.7	0.50
Interleukin-1 alpha (IL-1α)	[pg/ml]	143 ± 11	160 ± 13.4	0.49
Interleukin-1 beta (IL-1β)	[ng/ml]	3.9 ± 0.3	<3.5	0.14
Interleukin-2 (IL-2)	[pg/ml]	<49	54.3 ± 5.3	0.50
Interleukin-3 (IL-3)	[pg/ml]	<4.3	<4.3	NA
Interleukin-4 (IL-4)	[pg/ml]	<68	<68	NA
Interleukin-5 (IL-5)	[ng/ml]	0.8 ± 0.04	0.8 ± 0.004	0.39
Interleukin-6 (IL-6)	[pg/ml]	5.8 ± 2.0	<3.8	0.35
Interleukin-7 (IL-7)	[ng/ml]	<0.1	0.1 ± 0.003	0.44
Interleukin-10 (IL-10)	[pg/ml]	<220	<220	NA
Interleukin-11 (IL-11)	[pg/ml]	<70	<70	NA
Interleukin-12 Subunit p70 (IL-12p70)	[ng/ml]	<0.14	<0.14	NA
Interleukin-17A (IL-17A)	[ng/ml]	<0,007	<0.007	0.35
Interleukin-18 (IL-18)	[ng/ml]	22.6 ± 0.4	22.6 ± 1.1	>0.99

Leptin	[ng/ml]	<0.29	0.31 ± 0.008	0.17
Leukemia Inhibitory Factor (LIF)	[pg/ml]	865.6 ± 53.3	841.6 ± 41.1	0.73
Lymphotactin (XCL1)	[pg/ml]	124.3 ± 27.4	178.7 ± 9.4	0.16
Macrophage Colony-Stimulating Factor-1 (M-CSF-1)	[ng/ml]	7.1 ± 0.3	6.4 ± 0.4	0.21
Macrophage-Derived Chemokine (MDC)	[pg/ml]	1,608 ± 220.4	1,488 ± 66.1	0.62
Macrophage Inflammatory Protein-1alpha (MIP-1α)	[ng/ml]	6.3 ± 0.4	6.3 ± 0.2	0.93
Macrophage Inflammatory Protein-1 beta (MIP-1β)	[pg/ml]	112 ± 18.4	97,6 ± 15.2	0.56
Macrophage Inflammatory Protein-1 gamma (MIP-1γ)	[ng/ml]	16.8 ± 1.5	15 ± 0.9	0.35
Macrophage Inflammatory Protein-2 (MIP-2)	[pg/ml]	36.6 ± 3.9	33 ± 3.4	0.50
Macrophage Inflammatory Protein-3 beta (MIP-3β)	[ng/ml]	4.1 ± 0.1	3.8 ± 0.08	0.13
Matrix Metalloproteinase-9 (MMP-9)	[ng/ml]	39.4 ± 3.5	31.4 ± 1.1	0.06
Monocyte Chemotactic Protein 1 (MCP-1)	[pg/ml]	104.4 ± 16.0	78.2 ± 6.2	0.17
Monocyte Chemotactic Protein 3 (MCP-3)	[pg/ml]	255.4 ± 38.5	201.8 ± 19.2	0.25
Monocyte Chemotactic Protein-5 (MCP-5)	[pg/ml]	19.2 ± 2.7	15 ± 1.4	0.20
Myeloperoxidase (MPO)	[ng/ml]	42.8 ± 7.0	36.4 ± 3.2	0.43
Myoglobin	[ng/ml]	1,698 ± 160.9	1,430 ± 172.4	0.29
Oncostatin-M (OSM)	[ng/ml]	<0.2	0.3 ± 0.1	0.50
Plasminogen Activator Inhibitor 1 (PAI-1)	[ng/ml]	0.4 ± 0.1	0.3 ± 0.1	0.61
Resistin	[ng/ml]	<0.08	<0.08	NA
Serum Amyloid P-Component (SAP)	[µg/ml]	29.2 ± 0.2	33.6 ± 2.3	0.09
Stem Cell Factor (SCF)	[ng/ml]	637.8 ± 104.8	606 ± 92.7	0.83
T-Cell-Specific Protein RANTES (RANTES)	[ng/ml]	0.07 ± 0.01	0.07 ± 0.01	0.80
Thrombopoietin	[ng/ml]	103.6 ± 6.0	94.6 ± 5.0	0.28
Tissue Inhibitor of Metalloproteinases 1 Mouse (TIMP-1)	[ng/ml]	1.7 ± 0.3	1.9 ± 0.1	0.36
Tumor Necrosis Factor alpha (TNF-α)	[ng/ml]	<0.1	<0.1	NA
Vascular Cell Adhesion Molecule-1 (VCAM-1)	[ng/ml]	816.2 ± 71.4	735.2 ± 20.5	0.31
Vascular Endothelial Growth Factor A (VEGF-A)	[pg/ml]	271.4 ± 47.1	283.5 ± 44.6	0.86
von Willebrand factor (vWF)	[ng/ml]	180.6 ± 13.1	178.4 ± 14.1	0.91

Supplementary Table S2: Raw neurosphere counts of DG-derived neurosphere cultures treated with XCL1. Related to Figure 1c.

	Control	10 ng/ml	100 ng/ml
Experiment 1	57	99	103
Experiment 2	35	42	41
Experiment 3	77	87	122
Experiment 4	57	106	83
Experiment 5	92	122	Not tested
Experiment 6	36	39	Not tested
Experiment 7	37	56	Not tested
Experiment 8	35	43	40
Experiment 9	109	89	75
Experiment 10	51	135	144
Experiment 11	44	Not tested	75
Experiment 12	55	Not tested	62

Supplementary Table S3: Raw neurosphere counts of SVZ-derived neurosphere cultures treated with XCL1. Related to Figure 1d.

	Control	10 ng/ml	100 ng/ml
Experiment 1	590	1072	826
Experiment 2	226	260	291
Experiment 3	267	365	362
Experiment 4	503	562	690
Experiment 5	494	611	599
Experiment 6	440	520	540
Experiment 7	983	1318	Not tested
Experiment 8	666	884	Not tested
Experiment 9	795	866	Not tested

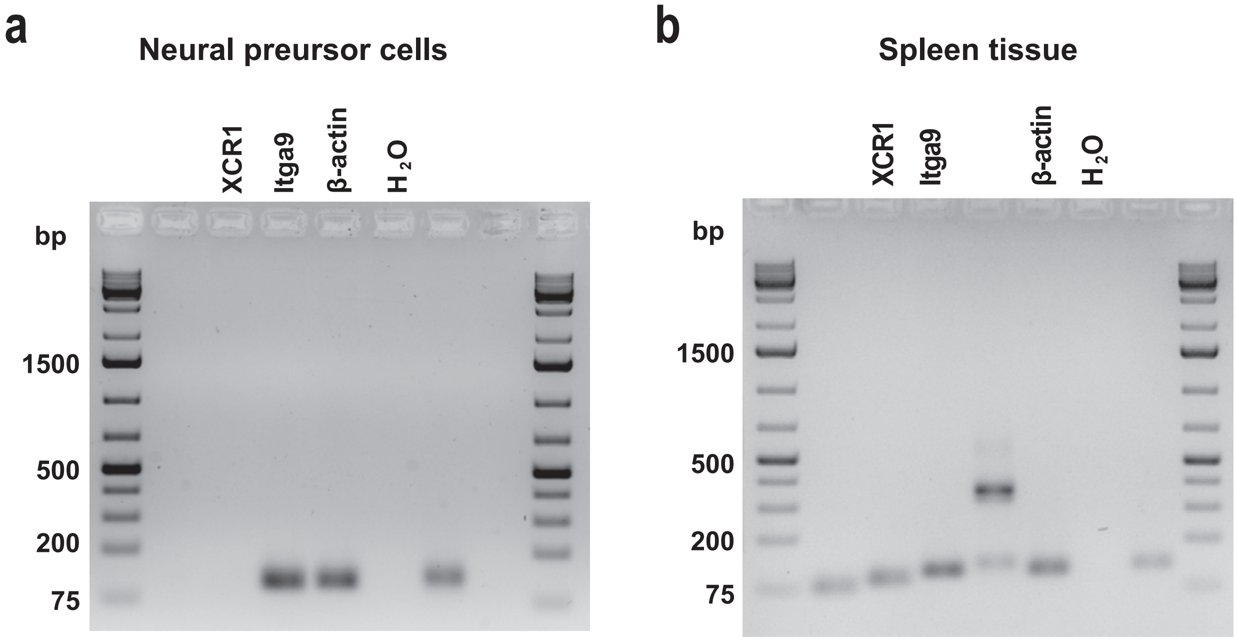

Supplementary Fig. S1: Gene expression analysis of lymphotactin receptors in neural precursor cells. ***a,*** qPCR gene expression analysis of the lymphotactin receptors XCR1 and Itga9 reveals that neural precursor cells express *Itga9* but not *Xcr1*. ***b,*** Both *Itga9* and *Xcr1* are detected in splenic control tissue. Electrophoretic gel images are uncropped with only relevant lanes labeled.

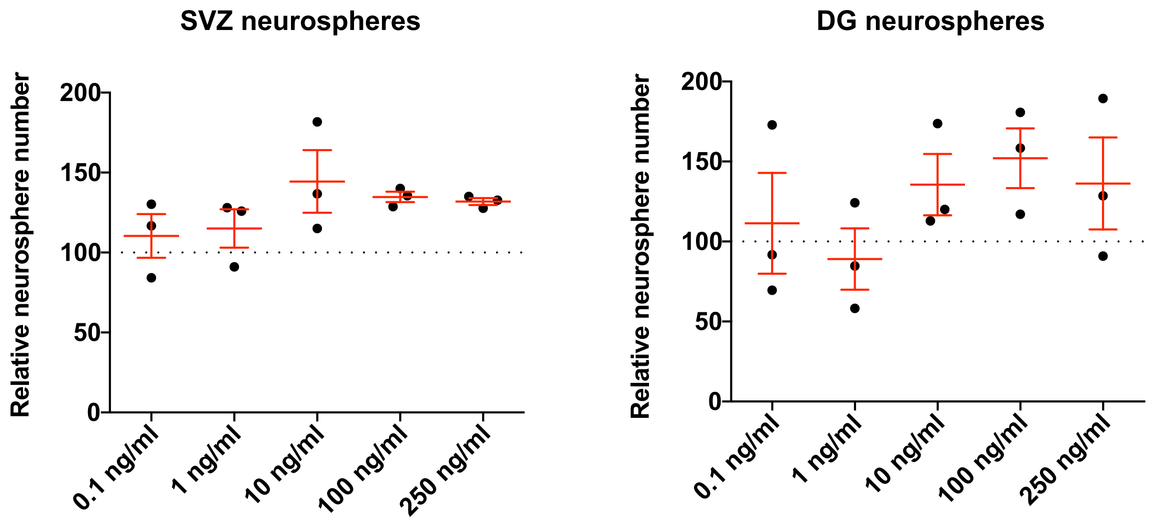

Supplementary Fig. S2: Neurosphere assay dose response experiments to test whether NPCs respond to XCL1 treatment. Different concentrations of XCL1 were tested on neural precursor cells derived from the SVZ and the DG. The most effective doses were determined to be 10 ng/ml and 100 ng/ml, both showing a trend towards a robust increase in neurosphere number after three independent neurosphere assay experiments.
